# Intervention Effects of Deer-Tendon Collagen Hydrolysates on Osteoporosis In Vitro and In Vivo

**DOI:** 10.3390/molecules28176275

**Published:** 2023-08-28

**Authors:** Chaoting Wen, Dan Wang, Zhiyi Zhang, Guoyan Liu, Li Liang, Xiaofang Liu, Jixian Zhang, Youdong Li, Xin Xu

**Affiliations:** College of Food Science and Engineering, Yangzhou University, Yangzhou 225127, China; chaoting@yzu.edu.cn (C.W.);

**Keywords:** collagen protein hydrolysates, degree of hydrolysis, intervention osteoporosis activities, MC3T3-E1, mouse tail-suspension test

## Abstract

Deer tendon, a deer processing byproduct, is an excellent protein source for the preparation of peptides for improving osteoporosis by its high protein content and high nutritional value. The optimal process of collagen acid extraction was implemented and the results showed that the acid concentration was 7%, the material–liquid ratio was 1:25, and the soaking time was 48 h. DTCHs could promote MC3T3-E1 cell proliferation and increase alkaline phosphatase activities in vitro. In addition, compared with the model group, the DTCHs treatment groups with an oral dosage of 350, 750, and 1500 mg/kg rat/day could significantly improve the shape, weight, bone mechanics, and alkaline phosphatase activities of tail-suspended mice. Bone microstructure and mineralization also recovered significantly in vivo. This result is expected to provide the structural and biological information for DTCHs-based functional foods.

## 1. Introduction

Osteoporosis is a common metabolic bone disease in China. It is expected that the incidence rate will increase to 14 million in 2020. Large numbers of studies have reported that collagen hydrolysate from animal byproducts could significantly improve the health of the body in osteoporosis interventions [1]. Deer tendon is one of the byproducts of deer processing. It is rich in a high proportion of collagen (82.12%), which can be used as a high-quality source of protein hydrolysate [2]. The collagen content of deer tendon was more than fish skin (21.6–21.95%) [3], pig skin (46%), bovine hide (29.4%), and pork and cattle bones (23.1%) [4], which showed stronger application prospects. Collagen hydrolysates are mainly used as functional substances in the processing of beverages [5], meat products [6], and diet-supplement products [7], among others. Among them, deer-tendon collagen hydrolysates (DTCHs) exhibited a strong ability to improve osteoporosis by increasing the proliferative activities and extracellular matrix synthesis of MC3T3-E1 cells [8].

In general, MC3T3-E1 cell models are widely used to evaluate the osteoporosis capacity of collagen hydrolysates. Although this method has the merits of easy operation and low cost, it cannot reflect the real situation of the human body after ingesting collagen hydrolysates. Therefore, it is important to establish an effective animal model to evaluate the intervention in osteoporosis of collagen hydrolysates in vivo. Animal models simulating osteoporosis mainly include an ovariectomized female mice model, a drug-induced osteoporosis model, and a mice tail-suspension test [9]. The ovariectomized female mouse model has the disadvantages of difficult operation and high cost. Drug-induced osteoporosis has many disadvantages, such as large side effects and unstable effects [9]. Compared with other models, the mouse tail-suspension test has the characteristics of short modeling time, remarkable effect, and stable effect [10]. To the best of our knowledge, the mechanism by which DTCHs are used to intervene in osteoporosis in tail-suspended mice is still unclear. Therefore, it is necessary to evaluate the effect of DTCHs on the intervention of osteoporosis in tail-suspended mice.

Therefore, the objective of this study was to (1) optimize the effect of the acid extraction process (acid concentration, material–liquid ratio, and soaking time) on collagen extraction capacities, which can be determined by using the deer-tendon dissolution time as an index; (2) study the hydrolysis degree and molecular weight distribution of DTCHs treated by enzymatic hydrolysis time (6 h, 12 h, 24 h, 36 h, and 48 h); (3) evaluate the activities of promoting the MC3T3-E1cell proliferation of DTCHs (the optimal hydrolysis efficiency group), which can be measured by cell viability and alkaline phosphatase activity; and (4) investigate the osteoporosis intervention of DTCHs (the optimal hydrolysis efficiency group) on tail-suspended mice by determining the shape, weight, bone mechanics, alkaline phosphatase activities, bone microstructure, and mineralization in vivo. This study is expected to develop new functional substances of collagen that can intervene in osteoporosis.

## 2. Results and Discussion

### 2.1. Optimization of Pretreatment of Acid-Soaked Deer Tendon

**Figure 1:**
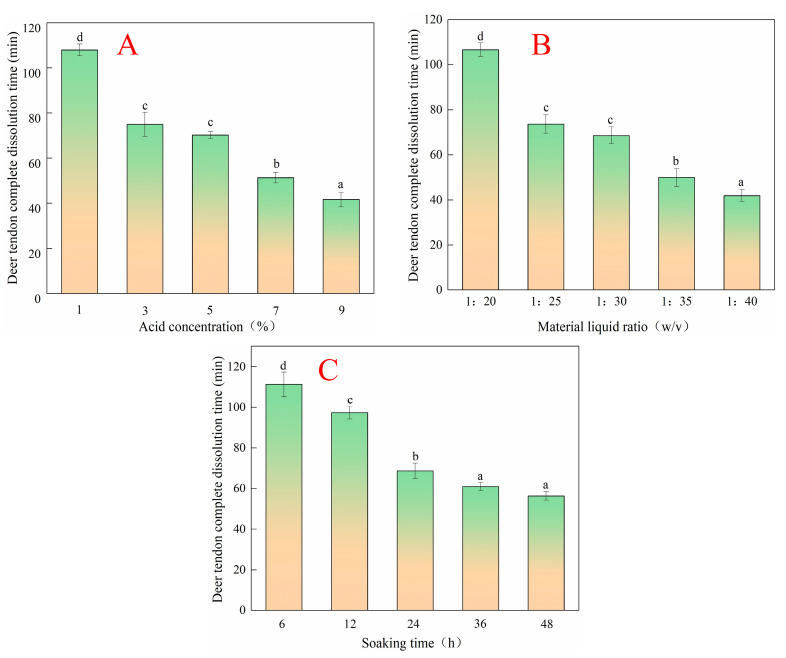
Effects of acid concentration, material–liquid ratio, and soaking time on the deer tendon complete dissolution time ((**A**): acid concentration; (**B**): material–liquid ratio; (**C**): soaking time). The different lowercase letters mean that the variance of different samples is significant (*p* < 0.05). Vertical bars indicate mean values ± SD (n = 3).

As reported by Cao et al. [11], citric acid treatment can significantly improve collagen yield (6%) and properties (elastic modulus, gelation, and melting temperature). Therefore, citric acid was used to extract collagen from deer tendons. The acid concentration, material–liquid ratio, and soaking time are routinely thought of as the important effect factors of the time for deer tendons to be completely dissolved in hot water. As shown in Figure 1A, there was a continuous decrease in deer tendon complete dissolution time when the acid concentration was ranging from 1% to 9%. This might be due to acid pretreatment, which could disrupt the stability of collagen molecular crosslinking, leading to the loss of ordered structure and promoting its dissolution [12]. Similarly, as shown in Figure 1B,C, there was a continuous decrease in deer tendon complete dissolution time when the material–liquid ratio and soaking time were 1:20–1:40 (*w*/*v*) and 6–48 h, respectively. This finding was consistent with the report of Skierka and Sadowska [13]; the longer the extraction time, the greater the solubility of collagen in the acid, which was due to the hydration of collagen fibers through the acid treatment. 

Considering the excessively high acid concentration, the material–liquid ratio and soaking time can increase the production cost. Meanwhile, multiple ionizable groups in citric acid could ionize a large number of active hydrogen ions at low concentrations, which destroyed the cross-linking of collagen molecules and improved the extraction rate of collagen protein [14]. The appropriate acid concentration (3%, 5%, 7%), material–liquid ratio (1:25, 1:30, 1:35 *w*/*v*), and soaking time (24, 36, 48 h) were selected for subsequent orthogonal test optimization. As shown in Table 1, range analysis showed that the order of factors affecting the complete dissolution time of deer tendon was A (acid concentration) > B (material–liquid ratio) > C (soaking time). The results of the analysis of variance indicated that the acid concentration had a significant effect on the complete dissolution time of the deer tendon, while the material–liquid ratio and soaking time had no significant effect on the complete dissolution time of the deer tendon (Table 1). Therefore, the optimal acid pretreatment process is A2B2C3.

The optimum process was verified by an orthogonal experiment. As Table 2 shows, sample 1 was as follows: acid concentration 7%, material–liquid ratio 1:25, and soaking time 48 h, whose complete dissolution time was 41.67 min. Meanwhile, sample 2 had an acid concentration of 7%, a material–liquid ratio of 1:35, and a soaking time of 48 h, which required 40.67 min for complete dissolution. There was no significant difference between the two samples but the amount of acid in sample 1 was less, which could reduce the production cost and salt generation. Therefore, the acid concentration of 7%, material–liquid ratio of 1:25, and soaking time of 48 h were the best acid pretreatment processes for deer-tendon collagen.

### 2.2. Effects of Different Enzymatic Hydrolysis Times on the Hydrolysis Efficiency of Deer-Tendon Collagen

**Figure 2:**
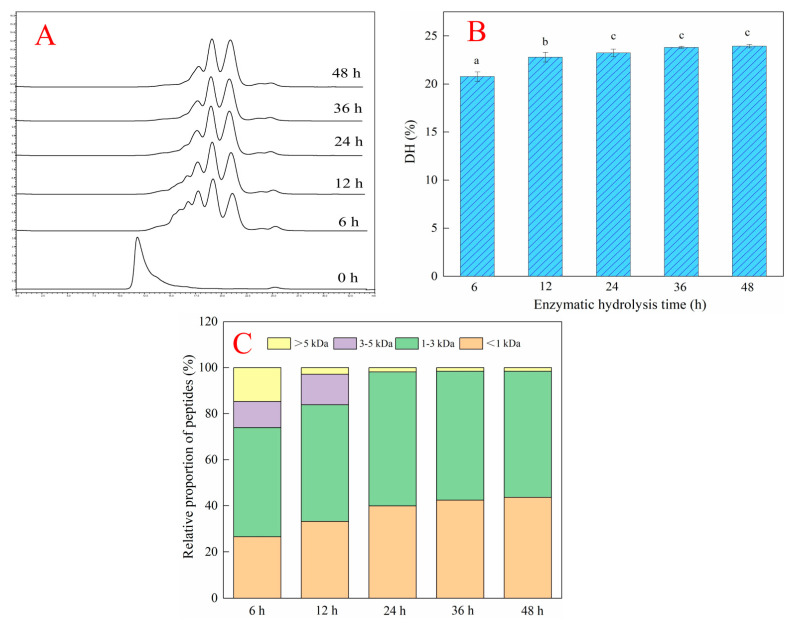
Effects of different enzymatic hydrolysis times on the hydrolysis efficiency of deer-tendon collagen ((**A**): gel chromatogram of molecular weight distribution; (**B**): degree of hydrolysis; (**C**): relative proportion of peptides with different molecular weights). The different lowercase letters mean that the variance of different samples is significant (*p* < 0.05). Vertical bars indicate mean values ± SD (n = 3).

Gel permeation chromatography can separate peptides according to their molecular weight. The larger the molecular weight of the peptides, the sooner they are separated. The smaller the molecular weight of tshe peptides, the later they are separated. As shown in Figure 2A, the peak time of DTCHs (enzymatic hydrolysis time: 6–48 h) was longer than that of collagen without enzymatic hydrolysis, which indicated that the molecular weight of hydrolysates after enzymatic hydrolysis became small. In addition, the hydrolysis degree of collagen increased with the increase of enzymatic hydrolysis time (Figure 2B), which was similar to Zhou et al. [15], who found that the degree of hydrolysis gradually increased and thereafter reached a plateau as the hydrolysis time prolonged. The order of hydrolysis degree of collagen from small to large was DTCHs-6 h (20.77%) < DTCHs-12 h (22.80%) < DTCHs-24 h (23.24%) < DTCHs-36 h (23.81%) < DTCHs-48 h (23.94%). It is worth noting that there is no difference in the degree of hydrolysis of collagen hydrolysates obtained when the enzymatic hydrolysis time is 24 h, 36 h, and 48 h. The bioactivity of peptides is closely related to their degree of hydrolysis, molecular weight, and structure. As shown in Figure 2C, the content of peptides with a molecular weight less than 3 kDa in DTCHs from small to large was DTCHs-6 h (74.01%) < DTCHs-12 h (83.85%) < DTCHs-24 h (98.13%) < DTCHs-36 h (98.46%) < DTCHs-48 h (98.43%). This trend was consistent with the degree of hydrolysis, due to the molecular weight distribution being highly correlated with the degree of hydrolysis [16]. Moreover, there is no difference in low molecular weight peptide content of collagen hydrolysates obtained when the enzymatic hydrolysis time is 24 h, 36 h, and 48 h. Thus, DTCHs-24 h was selected for the subsequent analysis.

### 2.3. Effects of Deer-Tendon Collagen Hydrolysates on the Proliferation of MC3T3-E1 Cells

**Figure 3:**
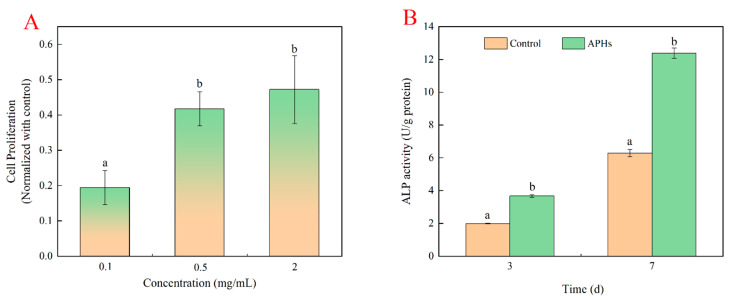
Effects of DTCHs on the proliferation of MC3T3-E1 cells ((**A**): cell proliferation; (**B**): ALP activities). The different lowercase letters mean that the variance of different samples is significant (*p* < 0.05). Vertical bars indicate mean values ± SD (n = 3).

DTCHs could promote the proliferation of MC3T3-E1 cells in a dose-dependent manner [8]. The MC3T3-El cell proliferation of DTCHs (0.1–2 mg/mL) were 0.06, 0.2, and 0.28, respectively (Figure 3A). In addition, the proliferation of osteoblasts treated with CPHs was significantly higher than that of collagen hydrolysates from fish, pig skin, and bovine at the same concentration [17]. Since there was little difference in the cell proliferation of MC3T3-El cells incubated with 0.5 mg/mL and 2 mg/mL of DTCHs, it was considered that 0.5 mg/mL DTCHs was used for the study of alkaline phosphatase activities. Similarly, Zhou et al. [8] pointed out that the enzymatic hydrolysates from deer tendon promoted MC3T3-E1 cell proliferation and extracellular matrix synthesis by regulating multiple functional genes.

The differentiation level of the osteoblasts was evaluated by measuring ALP activity, which was a differentiation marker at the early stage. Alkaline phosphatase played an important role in MC3T3-E1 cellular differentiation, mineralization, bone tissue formation, and hydrolyzed pyrophosphate [18]. As shown in Figure 3B, ALP activities of cells significantly increased in a time-dependent effect. The ALP activities of DTCHs were relatively low on day 3, where a value reached 3.67 ± 0.08 U/mg protein. However, ALP activities of DTCHs had a remarkable increase on day 7, when the value reached 12.38 ± 0.31 U/mg protein. This phenomenon was consistent with Liu et al. [19], who reported that collagen peptides from bovine played a positive role in the differentiation of MC3T3-E1 cells and significantly promoted the formation of mineralized bone matrix. 

In general, the trend of ALP activities of DTCHs was consistent with that of cell proliferation of DTCHs. This trend indicated that the proliferation capacity of cells could further promote cell differentiation by improving its ALP activities [18].

### 2.4. Intervention Effects of Collagen Protein Hydrolysates on Osteoporosis in Tail-Suspended Mice

#### 2.4.1. Analysis of Body Weight, Viscera Index, and Physiological Characteristics of Tail-Suspended Mice

**Figure 4:**
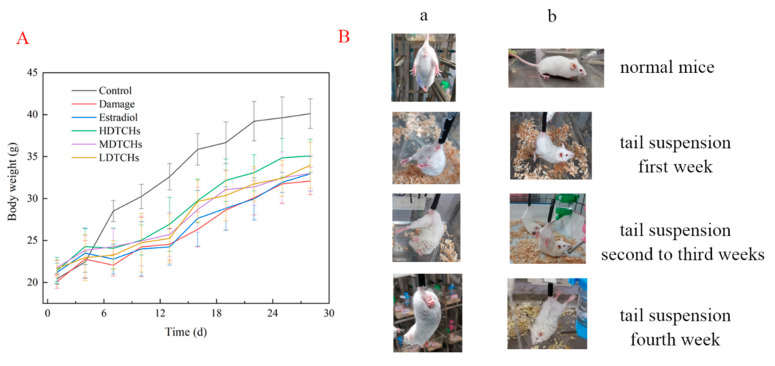
Effects of DTCHs on body weight and morphology of mice. (**A**): body weight; (**B**): morphology (Column a represents the changes in legs, column b represents the changes in states). Vertical bars indicate mean values ± SD (n = 3).

As shown in Figure 4A, there were no differences among different groups in body weight at the initial stage of tail suspension. Compared with the normal group, the body weight of the tail-suspended groups showed a decreasing trend after 7 days of tail suspension. The results were in good consistency with Yanagihara et al. [20], who reported that suspended rats had a smaller and slower body-mass gain than unrestricted rats. These results might be related to the loss of water electrolytes and appetite caused by the redistribution of body fluid under tail suspension [21]. 

Strikingly, the physical characteristics of mice were also affected by tail suspension (Figure 4B). The eyes of the mice were dim and the hind limbs were asymmetric after one week of tail suspension compared to the control. After four weeks of tail suspensions, the mice supported their bodies with their heads and their eyes closed. Some mice had their hind limbs contracted and hind paws folded. This might be because long-term suspension caused muscle atrophy, reduced muscle strength, bone stress, and traction on the bone, resulting in weakness and sagging of the hind limbs [22]. Meanwhile, the tail suspension could reduce bone mineral density and contribute to bone loss and bone quality decline.

In addition, the tail suspension could significantly reduce the index of all organs, especially the thymus and spleen, which were immune organs closely related to the body’s immune capacity (Table 3). This result suggested that suspension induced organ damage in mice, reducing their immunity, which might reduce the body weight of mice. However, with the oral administration of DTCHs, the indexes of the heart and spleen were significantly increased. The results indicated that DTCHs as a nutrient protects mouse organs from damage. Therefore, collagen hydrolysates could significantly protect mice with osteoporosis and improve their immune capacity.

The above results showed that tail suspension could reduce the body weight and worsen the physiological characteristics of mice, preliminarily indicating that the tail-suspension test was established successfully.

#### 2.4.2. Effects of Collagen Protein Hydrolysates on Serum and Heart Biochemical Indices in Tail-Suspended Mice

**Figure 5:**
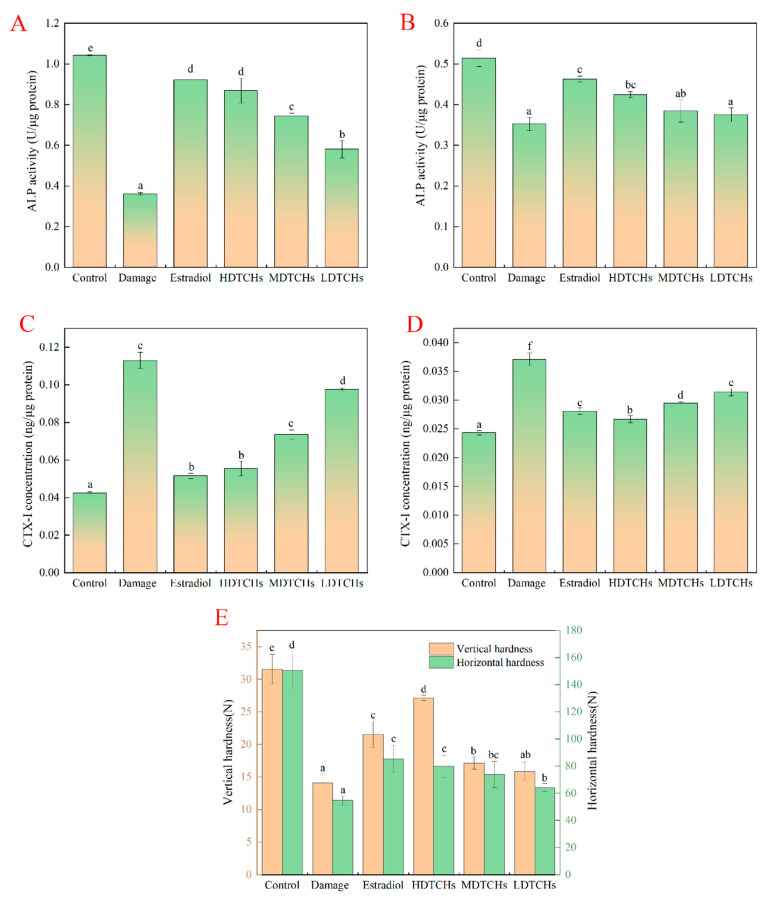
Effects of DTCHs on biochemical indexes and bone hardness of mice. ((**A**): ALP activity of mouse serum; (**B**): ALP activity of mouse heart; (**C**): CTX-I concentration of mouse serum; (**D**): CTX-I concentration of mouse heart; (**E**): Bone hardness of mice). The different lowercase letters mean that the variance of different samples is significant (*p* < 0.05). Vertical bars indicate mean values ± SD (n = 3).

Bone turnover in mice was evaluated by the levels of bone formation marker (ALP) and bone resorption marker (CTX-I) in the serum and the heart. ALP is a glycoprotein that anchors the cell membrane through hydrophobic glycosyl phosphatidylinositol and is the most commonly used biochemical marker for osteoblast bone formation, which could promote bone mineralization and facilitate bone formation by hydrolyzing phospholipids to release inorganic phosphorus and increase local phosphorus concentration [23]. CTX, as a special product produced by osteoclasts during bone resorption, is a nonhelical chain of cross-linked peptides at the end of type I collagen [24]. As shown in Figure 5A–D, compared with the control, ALP activity in the serums and hearts of mice after 4 weeks of suspension was significantly decreased, while the CTX-I concentration in serums and hearts was significantly increased. The results had good consistency with Jing et al. [25], whose results demonstrated that tail suspension could reduce the concentration of ALP, OC, and PINP in serum, inhibit osteoblasts’ activity, and increase the contents of TRAcP5b and CTX-I. Interestingly, compared to the damaged, the markers of bone transformation after DTCHs treatment were significantly improved, indicating that DTCHs could promote bone formation, inhibit bone resorption, and maintain the dynamic balance of bone transformation. Similar findings were also reported by Liu et al. [21]. However, in accordance with the study reported by Zhang et al. [26], compared to the control, the activity of ALP in ovariectomized mice was significantly increased and decreased after treatment with collagen protein hydrolysates, which may be caused by the differences between osteoporosis models.

The evaluation of bone biomechanical properties is indispensable to determine the quality of bone, and bone hardness and bone strength were directly related to bone structure. As shown in Figure 5E, a significant decrease in hardness was observed in the femora of tail-suspended mice. Measurement of vertical hardness from the femoral showed a 0.53-fold decrease after 4 weeks of tail suspension compared to the control. The DTCHs treatment prevented this decrease. The decrease in horizontal hardness in suspended rats was more pronounced (0.66-fold compared to unsuspended mice) and could not be prevented by DTCHs treatment (DTCHs-treated suspended rats showed a 0.5-fold decrease in horizontal hardness compared to control mice). The orders of vertical and horizontal hardness were: Control > HDTCHs > Estradiol > MATCHs > LDTCHs > Damaged, Control > Estradiol > HDTCHs > MATCHs > LDTCHs > Damaged, respectively. A similar phenomenon has been reported by Berg-Johansen et al. [27], who reported that microgravity could cause bone loss and decrease bone density, which might be that during bone resorption, osteoclasts formed a membrane on the bone matrix and secreted protons and hydrolases. Due to proton secretion, the lacuna was desalted under acidic conditions, resulting in the degradation of bone organic components by hydrolases, which, in turn, leads to a decrease in bone strength [28]. In addition, collagen protein hydrolysates increased bone strength and hardness by increasing collagen protein formation in bones. Moreover, Hyp-containing peptides foster osteoblast activity, resulting in increased bone mineralization and the synthesis of organic bone components [29]. The content of Hyp in deer tendon was 9.3% [30], which is higher than that of fish (6.7–6.8%) [31], bovine tendon (8.2%) [32], and pig skin (8.0%) [33]. Therefore, DTCH could effectively stimulate bone formation and inhibit bone resorption, thus enhancing the bone hardness of tail-suspended mice.

#### 2.4.3. Effect of Collagen Protein Hydrolysates on Histological Analysis of Femur in Tail-Suspended Mice

**Figure 6:**
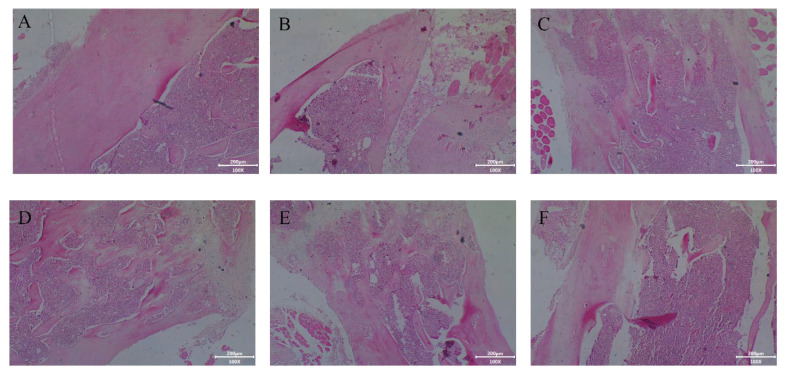
Effects of DTCHs on HE staining of mice tissue. (**A**): Control group; (**B**): Damaged group; (**C**): Estradiol group; (**D**): HDTCHs; (**E**): MDTCHs; and (**F**): LDTCHs.

Excessive bone resorption and lack of bone formation could cause reduced bone density and damage the bone microstructure and, finally, result in osteoporosis. Tail suspension induced a marked deterioration of femur microstructure in mice (Figure 6). Compared to the control, tail suspension could reduce the number of trabeculae and widen the bone marrow space. In line with Lam and Qin [34], who reported that the bone metabolic balance of tail-suspended mice was disordered, accompanied by bone trabecular loss. Meanwhile, tail suspension indicated a decrease in cortical bone thickness, implying that the potential fracture toughness was poor. These results suggested that real or simulated weightlessness could lead to bone changes and reduced bone strength and bone hardness (Figure 6E). Strikingly, DCTHs significantly inhibited the deterioration of bone microstructure and the reduction of cortical bone thickness in tail-suspended mice. However, the bone microstructure of mice treated with LDTCHs and MDTCHs could not be completely restored. HDTCHs exerted the best effect on normalizing the integrality of bone trabeculae, which were arranged in an orderly way and formed a network structure. 

The current study demonstrates that DTCHs were capable of preventing total trabecular and cortical bone deterioration. The tail suspension led to a decrease in bone hardness, the degeneration of bone trabeculae and cortical bone, and the increase of void area in bone marrow, which might be caused by the increase of stromal cells with osteoclastic potential [35]. Similarly, Machwate et al. [36] suggested that reduced bone formation was due to reduced proliferation of osteogenic precursor cells or bone matrix synthesis caused by tail suspension. Moreover, in the bone loss caused by tail suspension, proinflammatory cytokines had a stronger influence on the activity of osteoclasts than on osteoblasts; thus, the inhibition of bone formation was dominant, rather than accelerating bone resorption [35]. Collagen hydrolysates were rich in glycine and hydroxyproline, with strong antioxidant activity in vitro and in vivo, which were widely used in the medical and food fields [37]. Collagen protein hydrolysates from cod bone affected osteoclast activity and reduced bone loss by regulating the expression of RANKL and OPG and inhibiting the release of inflammatory factors [38]. In addition, Yang et al. [39] found that collagen protein hydrolysates from Atlantic cod significantly reduced receptor activator of nuclear factor (RANKL)-induced tartrate-resistant acid phosphatase (TRAP) activity by inhibiting the activation of MAPK and NF-κB pathways, thereby inhibiting osteoclast formation and bone resorption as well. Therefore, it could be concluded from the results that collagen protein hydrolysates could effectively reduce bone loss and improve bone morphology.

#### 2.4.4. Effect of Collagen Protein Hydrolysates on Femur Mineralization in Tail-Suspended Mice

**Figure 7:**
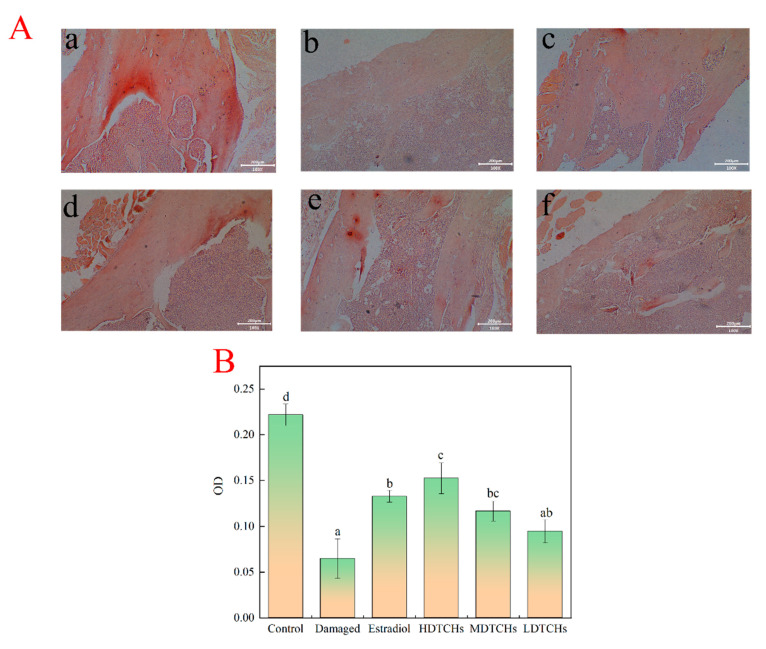
(**A**): Effects of DTCHs on alizarin staining of mice tissue (a: Control group; b: Damaged group; c: Estradiol group; d: HDTCHs; e: MDTCHs; f: LDTCHs); (**B**): Effects of DTCHs on image analysis of mouse femur stained with Alizarin. The different lowercase letters mean that the variance of different samples is significant (*p* < 0.05).

However, in addition to bone microstructure, characterizing changes in the degree of bone mineralization is important to enable an adequate and more specific analysis of bone loss after tail suspension. The number of calcium nodules in bone was determined by chromatin staining to reflect the degree of bone mineralization. The larger the dark red area, the higher the degree of bone mineralization. Compared to the control, the red area in the damaged group was the smallest, indicating that tail suspension significantly inhibited bone mineralization (Figure 7A). This result was consistent with Kawao et al. [40], whose results demonstrated that the tail suspension could inhibit the production of mineralized bone nodules with less osteoblast differentiation by increasing Dkk2 expression to induce RANKL expression. Moreover, Morey-Holton and Globus [41] believed that a decrease was found in bone mineralization in cortical and cancellous bones compared to the control, which could be that tail suspension resulted in delayed bone growth and reduced bone formation rate. According to the results of a bone mineralization image analysis, the DTCHs significantly increased the mineral deposition level in the femur in a concentration-dependent manner (Figure 7B). Compared with the damaged group, the HDTCHs showed a 1.6-fold increase in the degree of bone mineralization, which suggested that DTCHs can promote bone mineralization and prevent the loss of minerals in the bone. This might be because DTCHs were rich in peptides with a molecular weight < 3000 Da and collagen protein hydrolysates could increase the contents of various Hyp of oligopeptides in vivo, promoting alkaline phosphatase activity and bone mineralization. Heo et al. [42] found that collagen protein hydrolysates from fish bone interacted with the core interface residues in bone morphogenetic protein receptors with high affinity, which promote the proliferation of MC3T3-E1, alkaline phosphatase activity, and mineral deposition. In addition, collagen protein hydrolysates could accelerate mineralization by accelerating mineralization initiation and, subsequently, lead to increased calcium deposition via inducing COL1A1 (collagen, type I, alpha 1), ALPL (alkaline phosphatase), and SPP1 gene expression (secreted phosphoprotein 1, osteopontin) [43].

The above results showed that DTCHs could promote the proliferation and differentiation of MC3T3-E1, improve bone micromorphology, promote bone mineralization, inhibit the destruction of bone balance, and reduce the occurrence of osteoporosis.

## 3. Materials and Chemicals

### 3.1. Materials and Reagents

The deer (species: deer genus in the deer family; Full name: *Cervus nippon*) tendon was purchased from Huaguang Deer Industry Co., Ltd. (Yangzhou, China). MC3T3-E1 cells were obtained from Nanjing Jiancheng Biological Co., Ltd. (Nanjing, China). The 3-(4,5-dimethylthiahiazo-2-y1)-2,5-diphenyl tetrazolium bromide (MTT) and alkaline phosphatase (ALP) activity diagnostic kit were obtained from Nanjing Jiancheng Biochemistry Co., Ltd. (Nanjing, China). Alcalase (activity: 160,000 U/mL) was purchased from Novozymes Biotechnology Co., Ltd. (Tianjin, China). Six-week-old female KM mice (20–22 g, SCXK(JING)2019-0010) were purchased from Beijing Sibeifu Biochemistry Co., Ltd. (Beijing, China). Animal quality certificate number is SYXK(SU)2017-0044. All other chemicals used in the experiments were of analytical grade.

### 3.2. Preparation of Deer-Tendon Collagen

The deer tendon was extracted by using hot boiling water after acid soaking. The time that deer tendons completely dissolved in acid needs to be optimized by an orthogonal design experiment, which mainly includes three factors (acid concentration, material–liquid ratio, and soaking time). The design of the single factor and orthogonal L9 (33) test experiment was shown in Table 4 and Table 5. 

### 3.3. Preparation of Deer-Tendon Collagen Hydrolysates

The enzymatic hydrolysis conditions of collagen were as follows: collagen concentration was 5% (*w*/*v*), the addition of Alcalase was 0.6% (*w*/*w*) to control the enzyme activity to 1000 U/g, the pH was 9.0, the temperature was 40 °C, and the enzymatic hydrolysis times were 6 h, 12 h, 24 h, 36 h, and 48 h, respectively. After the enzymatic hydrolysis reaction, the Alcalase was deactivated by using a boiling water bath at 100 °C for 10 min, centrifuged at 4000× *g* for 30 min, and the supernatant was collected, freeze-dried, and stored for subsequent experiments. The enzymatic hydrolysates of collagen obtained after enzymatic hydrolysis for 6 h, 12 h, 24 h, 36 h, and 48 h were named DTCHs-6 h, DTCHs-12 h, DTCHs-24 h, DTCHs-36 h, and DTCHs-48 h, respectively.

### 3.4. Molecular Weight Distribution and Degree of Hydrolysis of Collagen Hydrolysates

#### 3.4.1. Molecular Weight Distribution of Collagen Protein Hydrolysates

The molecular weight distribution of deer-tendon collagen hydrolysates (DTCHs) was measured based on the previous method [44] by using a high-performance liquid chromatography system with a TSK gel G2000 SWXL column (7.8 × 300 mm, Tosoh, Tokyo, Japan). The uridine (244.2 Da), oxytocin (1007 Da), insulin (5733 Da), and whey protein (14,176 Da) were used as standards to obtain the calibration curve.

#### 3.4.2. Determination of the Degree of Hydrolysis of Collagen Protein Hydrolysates

The degree of hydrolysis (DH) of DTCHs was evaluated by using the previous method [44] with slight modification. After mixing formaldehyde reagent (37%) and DTCHs in the ratio of 1:1 (*v*/*v*), 5 mL of NaOH (0.2 mol/L) solution and phenolphthalein were added as an indicator, the solution was titrated with HCl (0.2 mol/L) until the solution became colorless, and the consumed volume of NaOH and HCl solution was recorded. The calculation formula for the DH of DTCHs was as follows:

$$\mathrm{DH}\;\left(\%\right)=\frac{\alpha \text{-}\mathrm{amino}\;\mathrm{nitrogen}\;}{\mathrm{Total}\;\mathrm{nitrogen}\;}\times 100$$

### 3.5. Effects of Deer-Tendon Collagen Hydrolysates on the Proliferation of MC3T3-E1 Cells

MC3T3-E1 cells were cultured in the α-MEM medium (containing 10% fetal bovine serum and 1% penicillin-streptomycin) in a humidified incubator (5% CO_2_, 37 °C) for 24 h. The effects of DTCHs incubation on the MC3T3-E1 cell proliferation were measured by using the MTT method of Wen et al. [45]. The MC3T3-E1 cells (1 × 10^4^ cells/well) were seeded in 96-well plates after incubating for 24 h and the different concentrations of DTCHs (0.5 mg/mL, 1 mg/mL, and 2 mg/mL) were added into each well for 4 h incubation. Then, the culture medium was replaced by using dimethyl sulfoxide; after mixing for 10 min, the optical density of the cell was measured by using a microplate reader (Infinite M200 Pro, Becton, Houston, TX, USA) at the wavelength of 490 nm.

#### Evaluation of Alkaline Phosphatase Activity of Collagen Hydrolysates

Evaluation of alkaline phosphatase (ALP) activity of collagen hydrolysates was based on the method of ALP activity diagnostic kit (Nanjing, China). Briefly, the cells were digested by using trypsin at 3 and 7 days and the absorbance values of the ALP activity and the protein concentration of cells were measured by using a Microplate reader (Infinite M200 Pro, Becton, Houston, TX, USA). ALP activity was expressed in U/g protein.

### 3.6. Intervention Capacities of Collagen Hydrolysates on Osteoporosis in Tail-Suspended Mice

#### 3.6.1. Establishment and Experimental Grouping of Mice Tail-Suspension Test

The establishment of the mice tail-suspension test model was performed in accordance with the report by Yanagihara et al. [20] with minor modifications. In brief, the tail of the mouse was fixed on the transverse rod to keep its torso inclined at an angle of 30° to the ground (the hind limbs cannot touch the ground). Meanwhile, mice can have free access to food and water.

**Figure 8:**
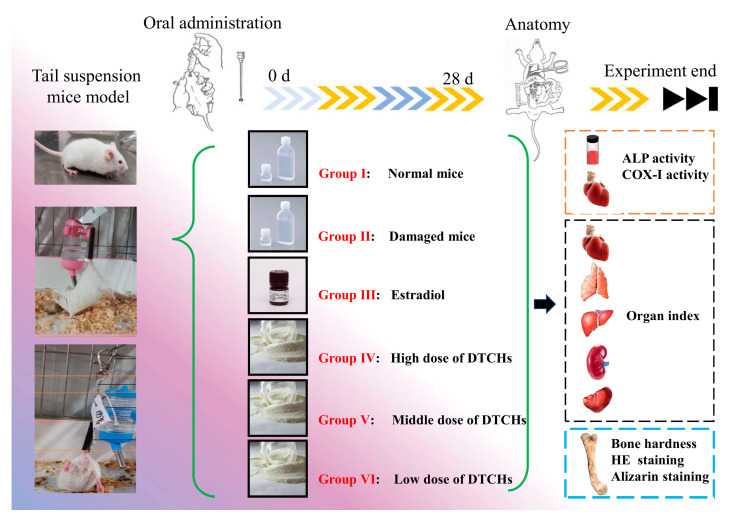
Flow chart of animal experiments.

The female KM mice were separated into six groups of eight mice in each group. The mice of the control group were orally administered with sterile water (Group I). The mice of the damaged group were suspended and orally administered with sterile water (Group II). The female KM mice of the positive control group were suspended and orally administered with estradiol (0.2 mg/kg rat/day) (Group III). The female KM mice were suspended and orally administered with DTCHs (1500 mg/kg rat/day) (Group IV). The female KM mice were suspended and orally administered with DTCHs (750 mg/kg rat/day) (Group V). The female KM mice were suspended and orally administered with DTCHs (250 mg/kg rat/day) (Group Ⅵ). KM mice were orally administrated once a day for 28 days. The flow chart for the animal experiments is illustrated in Figure 8. At the end of the experiment, the mice were sacrificed with Zoletil and CO_2_ and, then, the heart, liver, kidney, spleen, thymus, and femora were dissected and separated for subsequent experiments.

#### 3.6.2. Morphology, Body Weight, and Organ Index Determination of Mice

The mice were weighed and recorded every three days, and the morphological changes of the mice were observed. After 28 d, mice were sacrificed and the organs (heart, liver, kidney, spleen, and thymus) were collected for organ index analysis. The organ index was calculated based on the following equation [29]:

$$\mathrm{Organ}\;\mathrm{index}\;=\frac{\mathrm{Organ}\;\mathrm{weight}}{\mathrm{Body}\;\mathrm{weight}}\times \;100\%$$

#### 3.6.3. Determination of Bone Hardness in Mice

The horizontal and vertical hardness of the right femurs of mice were measured by a texture analyzer (TMS-Pro, Food Technology Co., Sterling, VA, USA) with an extrusion and shear probe. The measurement conditions were set as the minimum trigger force of 0.5 N, detection speed of 60 mm/s, and compression deformation of 70%.

#### 3.6.4. Determination of Alkaline Phosphatase Enzyme and Type I Collagen C-Terminal Telopeptide Enzyme Activities in Mice

The activities of ALP and C-terminal telopeptide of the type I collagen (COX-I) enzyme in the serums and hearts of mice were evaluated according to the manual of kits (Nanjing, China).

#### 3.6.5. Histological and Alizarin Staining Assay

The rat femoral tissues were decalcified, dehydrated, and embedded in paraffin. Then, the paraffin-containing rat femoral tissues were stained with hematoxylin and eosin (H&E) according to the method of Zhang et al. [29]. The changes in histology were observed by light microscopy at 100× magnification.

The bone mineralization degree was evaluated based on the alizarin staining method of Lievremont, Potus, and Guillou [46], with minor modifications. The paraffin-containing rat femoral tissues were sliced, dried, dehydrated, and stained with alizarin dye (2.5%) for 2 min. The changes in bone mineralization were observed by light microscopy at 100× magnification and the relative absorbance of alizarin staining was analyzed by Pro Plus 6.0 image analysis software.

### 3.7. Statistical Analysis

The statistical analysis was evaluated by using SPSS 18.0 software (SPSS Inc. Chicago, IL, USA). The significant differences between the two sets of data were analyzed by using Student’s T-test. Duncan’s Multiple Range test (DMRT) was executed as a post hoc test to find the significant impact of all treatments at a 5%-level of significance.

## 4. Conclusions

In summary, DTCHs, as collagen hydrolysates of deer tendon rich in small molecular peptides, have good antiosteoporosis activity in vivo and in vitro, which could prevent osteoporosis by stimulating MC3T3-E1 proliferation and differentiation to promote bone formation. In addition, DTCHs have been found to increase bone hardness, improve microstructure, enhanced bone mineralization, and reduce bone loss in tail-suspended mice. These findings provided a reasonable basis for the potential utility of DTCHs in the prevention and treatment of osteoporosis. The subsequent study will explore the effect of DTCHs on osteoclasts and fully clarify its specific mechanism of antiosteoporosis.

## Figures and Tables

**Table 1:** Results of the orthogonal experiment.

Samples	Acid Concentration (A)	Material–Liquid (B)	Soaking Time (C)	Index (min)
1	1	1	1	94.81 ± 1.56
2	1	2	2	70.26 ± 1.75
3	1	3	3	55.23 ± 1.08
4	2	1	2	60.04 ± 2.77
5	2	2	3	45.03 ± 0.75
6	2	3	1	64.84 ± 0.62
7	3	1	3	40.22 ± 1.51
8	3	2	1	49.82 ± 1.24
9	3	3	2	45.01 ± 1.40
K_1_	220.3	195.07	184.85	
K_2_	169.91	165.11	175.32	
K_3_	135.05	165.08	165.09	
k_1_	73.43	65.02	61.62	
k_2_	56.64	55.04	58.44	
k_3_	45.07	55.03	55.03	
R	28.36	9.99	6.59	
Sig.	*p* < 0.05	*p* > 0.05	*p* > 0.05	

Note: Vertical bars indicate mean values ± SD (n = 3). *p* < 0.05 represents the statistical significance.

**Table 2:** The results of the validation test.

Samples	Acid Concentration (A)	Material–Liquid (B)	Soaking Time (C)	Results (min)
1	7%	1:25	48 h	41.67 ± 1.53 ^a^
2	7%	1:35	48 h	40.67 ± 1.15 ^a^

Note: Different lower letters denote statistical significance (*p* < 0.05) difference between samples. Data are mean ± SD (n = 3).

**Table 3:** Viscera indexes of mice.

Samples	Cardiac Index (%)	Liver Index (%)	Renal Index (%)	Spleen Index (%)	Thymus Index (%)
Control	0.64 ± 0.05 ^c^	5.64 ± 0.18 ^b^	1.66 ± 0.17 ^a^	0.37 ± 0.03 ^d^	0.33 ± 0.04 ^c^
Damage	0.49 ± 0.09 ^a^	5.02 ± 0.26 ^a^	1.63 ± 0.13 ^a^	0.21 ± 0.01 ^a^	0.17 ± 0.04 ^a^
Estradiol	0.61 ± 0.01 ^bc^	5.72 ± 0.38 ^b^	1.64 ± 0.19 ^a^	0.28 ± 0.03 ^bc^	0.27 ± 0.04 ^bc^
HDTCHs	0.59 ± 0.03 ^bc^	5.50 ± 0.30 ^ab^	1.68 ± 0.14 ^a^	0.32 ± 0.02 ^c^	0.24 ± 0.06 ^ab^
MDTCHs	0.58 ± 0.06 ^bc^	5.42 ± 0.08 ^ab^	1.79 ± 0.17 ^a^	0.27 ± 0.05 ^bc^	0.19 ± 0.07 ^ab^
LDTCHs	0.55 ± 0.02 ^ab^	5.09 ± 0.20 ^a^	1.63 ± 0.16 ^a^	0.23 ± 0.03 ^ab^	0.18 ± 0.07 ^ab^

Note: Different lower letters denote statistically significant (*p* < 0.05) differences between samples. Data are mean ± SD (n = 3).

**Table 4:** Design of the single-factor experiment.

Levels	Factors		
	Acid Concentration (g/mL)	Material–Liquid Ratio (*w*/*v*)	Soaking Time (h)
1	3	1:25	24
2	5	1:30	36
3	7	1:35	48

**Table 5:** Design of the orthogonal experiment.

Samples	Acid Concentration (A)	Material–Liquid Ratio (B)	Soaking Time (C)
1	1	1	1
2	1	2	2
3	1	3	3
4	2	1	2
5	2	2	3
6	2	3	1
7	3	1	3
8	3	2	1
9	3	3	2

## Data Availability

Not applicable.
